# Navigating the structure of COMPASS

**DOI:** 10.7554/eLife.54767

**Published:** 2020-02-24

**Authors:** Karolin Luger, Jonathan W Markert

**Affiliations:** 1 Department of Biochemistry, University of ColoradoBoulderUnited States

**Keywords:** ubiquitin, histone methylation, chromatin, transcription, cryo-electron microscopy, *S. cerevisiae*

## Abstract

Cryo-electron microscopy reveals how ubiquitination promotes the methylation of histone H3 by the histone-modifying complex COMPASS.

**Related research article** Worden EJ, Zhang X, Wolberger C. 2020. Structural basis for COMPASS recognition of an H2B-ubiquitinated nucleosome. *eLife* **9**:e53199. doi: 10.7554/eLife.53199

Chromatin packages DNA by wrapping it around a core made of eight histone proteins to form individual units called nucleosomes which can be stored inside the nucleus (Luger et al., 1997). Each core contains four different types of histone, which each have flexible extensions called N-terminal tails. Certain modifications to these tails allow proteins to be recruited to the nucleosome, which can alter the structure of chromatin and make various regions of DNA more or less accessible. As a result, histone modifications regulate many genetic processes, including transcription, DNA repair and replication.

One modification that is particularly important for both transcription and repair is the addition of methyl groups to a lysine residue that sits within the N-terminal tail of histone H3 (Kouzarides, 2007). In yeast, methylation of this residue (also known as H3K4) relies on an enzyme called Set1 which forms part of the protein complex COMPASS (Figure 1A; Miller et al., 2001). Previous work has shown that for COMPASS to be recruited to the nucleosome, a ubiqutin group must be attached to a lysine residue in another histone called H2B (Kim et al., 2013). However, this lysine residue is far away from the N-terminal tail of H3: so why does COMPASS need H2B to be ubiquitinated in order to methylate this region?

**Figure 1.**
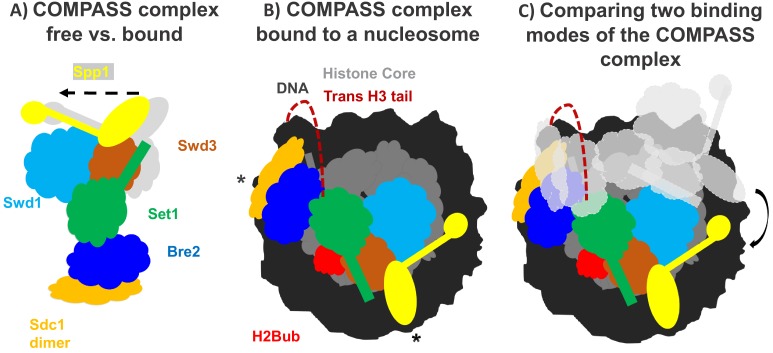
How the COMPASS complex binds to the nucleosome. (**A**) The COMPASS complex consists of six subunits including the enzyme Set1 (green) which methylates histone H3. When COMPASS binds to the nucleosome (not shown), three of its subunits (Swd3, Spp1 and Sd1) are slightly rotated towards the nucleosome (in the direction of the arrow; original positions shown in grey). (**B**) As well as rotating these three subunits, ubiquitination of the histone H2B (H2Bub; red) allows COMPASS to properly bind to the nucleosome and contact the DNA (black) at three distinct locations, including two which are at opposing ends of the nucleosome (indicated by *). Together these conformational changes help place the N-terminal tail of histone H3 (red dashed line) into the active site of Set1 so that H3 can be methylated. (**C**) Comparing COMPASS complexes which are bound to ubiquitinated (colored diagram) and non-ubiquitinated nucleosomes (light grey diagram) revealed that ubiquitin allows COMPASS to be in a better position for methylating the N-terminal tail of histone H3.

A common way to understand how histone modifying proteins interact with the nucleosome is to determine their structure using cryo-electron microscopy (Zhou et al., 2019; Worden et al., 2019). Now, in eLife, Evan Worden, Xiangbin Zhang and Cynthia Wolberger from Johns Hopkins University School of Medicine report how they used this technique to understand how COMPASS recognizes nucleosomes containing ubiquitinated H2B and uses this site to add a methyl group to histone H3 (Worden et al., 2020).

Worden et al. collaborated with another research group to develop a semi-synthetic H2B protein with a ubquitin chain attached to the correct lysine (Morgan et al., 2016). The team then used cryo-electron microscopy to produce a high-resolution structure of COMPASS from yeast that was bound to a nucleosome made with the semi-synthetic histone protein. This revealed that COMPASS spans the entire face of the nucleosome, making contacts with DNA at three distinct locations, two of which are at opposing edges of the nucleosome (Figure 1B).

The structure also showed that COMPASS makes extensive interactions with the histone core, molding itself over the surface of the nucleosome at the site where H2B binds together with another histone called H2A. Compared to structures of isolated COMPASS (Qu et al., 2018; Takahashi et al., 2011), three of the subunits in the bound complex were found to rotate towards the nucleosome and strengthen this interaction. This rotation also slightly shifts the position of the Set1 enzyme so that it can accept the H3 tail and allow methylation to occur (Figure 1A,B).

Worden et al. found that three of the COMPASS subunits cluster together to interact with the ubiquitin modification attached to the tip of H2B. This helps form a stronger attachment between COMPASS and H2B, and explains the preference for ubiquitinated nucleosomes. Additionally, this extensive interaction positions COMPASS so that the H3 tail can reach into the active site of the enzymatic subunit Set1. Although the tail of H3 is disordered as it emerges from the tightly-coiled DNA superhelix, it is still possible to see the tail tip sitting within the active site of COMPASS. This suggests that the N-terminal tail snakes around the outside of the nucleosome to reach the active site (Figure 1B,C). This could prohibit spontaneous conformational changes that expose regions of DNA (also known as DNA breathing) and affect the stability of the nucleosome.

Next, Worden et al. set out to find if the structural features predicted to be important for methylation had an effect in vivo. They found that introducing mutations which prevented COMPASS from interacting with the nucleosome, or which disrupted the interactions with the ubiquitin chains on H2B, led to reduced H3K4 methylation. These in vivo results represent a near-perfect match with the structural data and support this newly identified structure of COMPASS.

Other research groups have recently defined the structures of additional COMPASS complexes, including the human COMPASS complex MLL1 and a nucleosome-bound complex from a different strain of yeast (Park et al., 2019; Xue et al., 2019; Hsu et al., 2019). Out of the seven COMPASS structures determined, the three that were bound to ubiquitinated nucleosomes had nearly identical structures. The remaining four structures were bound to unmodified nucleosomes: whilst two of the structures had a similar conformation to COMPASS complexes bound to modified nucleosomes, in the other two structures COMPASS was rotated away from the histone core.

The structural differences between COMPASS proteins bound to modified and unmodified nucleosomes suggest that the ubiquitination of H2B (and the additional interactions with COMPASS) stabilizes the complex in a better position for promoting H3K4 methylation (Figure 1C). Taken together, these findings provide a new insight into how modifications across different histones can work together to regulate gene expression. Further work should focus on how the strength of the interaction between COMPASS and the ubiquitinated nucleosome influences the methylation of H3K4.
